# POCUS teaching - needs and reality based on 500 respondents

**DOI:** 10.1186/s12909-026-08860-1

**Published:** 2026-02-25

**Authors:** Marcelina Sadowska, Ana Segura, Elena Segura-Grau, Martin Altersberger, Natalia Buda

**Affiliations:** 1 Clinical Ultrasound Laboratory, Medical Simulation Center, Medical University of Gdansk, Gdańsk, Poland; 2 Centro Diagnóstico Ecográfico, Hospital San Francisco de Asis, Madrid, Spain; 3 Anesthesiologist Centro Hospitalar Tondela Viseu, ULS Viseu Dão Lafões, Viseu, Portugal; 4 Pyhrn-Eisenwurzen Klinikum, Steyr, Austria

**Keywords:** Ultrasound point-of-care, Sonography, Education

## Abstract

**Background:**

Ultrasonography, due to its numerous advantages, has become an indispensable tool in the daily work of physicians. However, there remains a limited body of knowledge regarding the current methods of ultrasound education among medical professionals. A survey was conducted among physicians to assess their previous training in Point-of-Care Ultrasound (POCUS) and their expectations regarding the timing and structure of such education.

**Methodology:**

The survey was randomly administered to a group of 500 practicing physicians from Poland, Austria, Spain, and Portugal. The questionnaire consisted of 10 items addressing the perceived usefulness of ultrasound in daily clinical practice, the respondents’ ability to perform ultrasound examinations and interventional procedures under ultrasound guidance, as well as their previous exposure to ultrasound training and expectations regarding the ideal time to initiate education in this area.

**Results:**

The survey revealed that current POCUS education during undergraduate and postgraduate internship training is inadequate. A total of 76.4% of respondents reported that their medical studies did not adequately prepare them to use ultrasound in clinical practice, and only 9.2% indicated that ultrasound training was a mandatory component of their university curriculum. The vast majority reported acquiring POCUS skills through self-initiated courses outside of their residency programs. Notably, 53.8% of respondents expressed a need for ultrasound education to begin during medical school, while 96.6% stated that the ability to perform ultrasound examinations would significantly improve their clinical work.

**Conclusion:**

There is a strong and clearly expressed demand for POCUS education to be introduced at an early stage of medical training. Ultrasound should be integrated as a mandatory component of the medical curriculum, as it greatly facilitates clinical decision-making and enhances the quality of patient care.

**Supplementary Information:**

The online version contains supplementary material available at 10.1186/s12909-026-08860-1.

## Background

Ultrasonography, owing to its increasing accessibility, has become an indispensable tool in the daily practice of physicians. It is a non-invasive and rapid procedure that effectively complements the physical examination of patients [1, 2]. Today, the availability of ultrasound devices is vastly greater than it was just a few decades ago. A wide range of portable ultrasound machines are now available, some compact enough to fit into a physician’s coat pocket and operable via wireless probes and smartphones. These modern portable devices, which often include Doppler imaging capabilities, are increasingly affordable and widespread among clinicians working in various healthcare settings [3, 4].

In line with the concept of point-of-care, the examination is performed at the patient’s bedside and is intended to answer specific clinical questions posed by the clinician [5]. Despite numerous reports highlighting the utility of ultrasonography in emergency medicine, pediatrics, intensive care, and internal medicine [6–8], there remains a significant gap in the literature regarding the methods used to teach ultrasound to medical professionals. Medical school curricula, aside from traditional radiology courses, rarely offer dedicated and regular instruction focused on the development of point-of-care ultrasound (POCUS) skills.

Analysis of data from Portugal, Spain, and Austria reveals that ultrasound curricula are inconsistent and often integrated into various subjects such as radiology, cardiology, and obstetrics, with relatively few hours devoted to ultrasound training. According to survey respondents, very few medical schools offer dedicated courses in ultrasonography. Similar findings were reported in a Canadian survey, where only 50% of medical schools included ultrasound training in their curricula, with programs varying widely and offering an average of only 5 h of instruction per year, which is deemed insufficient [9].

The World Federation for Ultrasound in Medicine and Biology (WFUMB) has identified several persistent barriers that limit the implementation of ultrasound education in medical curricula worldwide. These include overloaded curricula, where limited teaching time competes with numerous core subjects; restricted access to ultrasound equipment, which limits hands-on training opportunities; and a shortage of qualified instructors, which remains one of the most significant obstacles to developing structured POCUS teaching programs. WFUMB emphasizes that overcoming these barriers requires the adoption of innovative educational methods such as near-peer teaching, simulator-based training, and the integration of e-learning resources, which can optimize available time and resources while improving learning efficiency [10].

The primary objective of this study was to analyze the educational pathways of physicians in the field of ultrasonography through the use of a survey. The questionnaire aimed to explore participants’ expectations regarding ultrasound education and to compare these with the actual training experiences they encountered during their professional development.

## Methodology

The survey was conducted randomly among a group of 500 actively practicing medical professionals. Responses were collected from physicians across various specialties in four European countries: Austria, Spain, Germany, and Portugal. The participants were randomly selected from actively practicing physicians in four European countries (Poland, Austria, Spain, and Portugal). The survey was distributed through mailing lists of scientific societies and professional organizations, ensuring a random selection of respondents representing various specialties and stages of professional development. The questionnaire did not differentiate based on the stage of career development, both residents and fully trained specialists from various medical disciplines were included in the study. The questionnaire was developed based on previously published POCUS education frameworks and authors’ consensus. It consisted of 10 multiple-choice questions addressing educational background, self-assessed competency, and preferred timing for ultrasound education. The questionnaire addressed several key domains: the timing and circumstances of the respondents’ first exposure to ultrasound and level of preparedness to perform POCUS during medical school. Additionally, the questions asked respondents of preferred stage of education for learning ultrasound skills and the frequency of ultrasound use in daily clinical work and the perceived value of basic ultrasound competence in improving clinical decision-making. An important question that highlighted a significant gap in current medical education concerned the ability to perform interventional procedures under ultrasound guidance. The primary aim of the survey was to confront the participants’ real-world experiences in ultrasound training with their expectations concerning the optimal model of education in this area. All 500 responses included in the analysis were fully completed, no partial responses were excluded. 

The main survey was supplemented with an additional questionnaire completed by medical professionals in Poland, which focused on the teaching methods employed during commercial ultrasound training courses (Table 1).

**Table 1:** Characteristics of respondents by country

	Austria	Spain	Poland	Portugal
Total number of participants	70	218	127	85
Residents	36	121	56	50
Specialists	34	97	71	35
Most represented specialties	Internal Medicine, Anesthesiology, Family Medicine	Family Medicine, Anesthesiology, Emergency Medicine	Family Medicine, Internal Medicine, Emergency Medicine	Anesthesiology, Internal Medicine, Family Medicine

## Results

The majority of participants were family medicine physicians (31.7%) and internal medicine specialists (23%), including nephrologists, cardiologists, gastroenterologists, and pulmonologists. The next largest group comprised anesthesiologists and intensivists (21.6%). The remaining respondents were specialists in fields such as surgery (11%), emergency medicine (10.7%), and pediatrics (2.2%).

In the study group, 47.4% of the respondents were specialists, while 52.6% were residents. This distribution reflects a balanced representation of both groups, allowing for the assessment of perspectives from physicians at different stages of professional training.

The first question concerned the timing of the respondents’ first opportunity to perform an ultrasound examination independently.

**Fig. 1:**
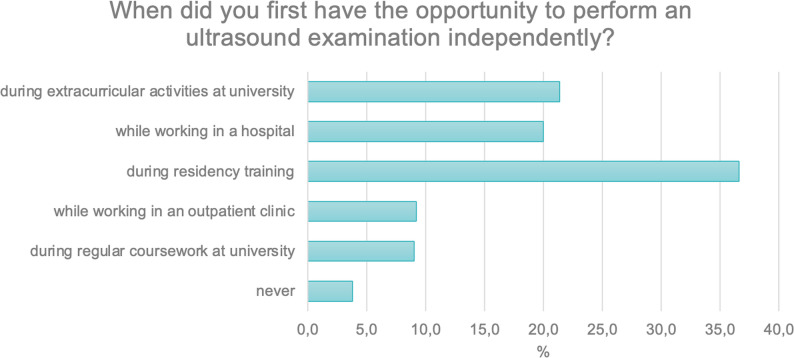
Timing of the first independent ultrasound examination among respondents

Approximately 20% of participants gained this experience during their studies as part of extracurricular activities (*N* = 107; 21.4%), during their specialty training (*N* = 102; 20.4%), or while working in a hospital setting (*N* = 100; 20.0%). A further 16.2% (*N* = 81) reported attempting ultrasound examinations during their residency training. About 9% of respondents performed ultrasound examinations in outpatient practice (*N* = 46; 9.2%) or during regular university classes (*N* = 45; 9.0%) (Fig. 1).

In the next question, respondents were asked about their preparation for performing point-of-care ultrasound (POCUS) during their university studies.

**Fig. 2:**
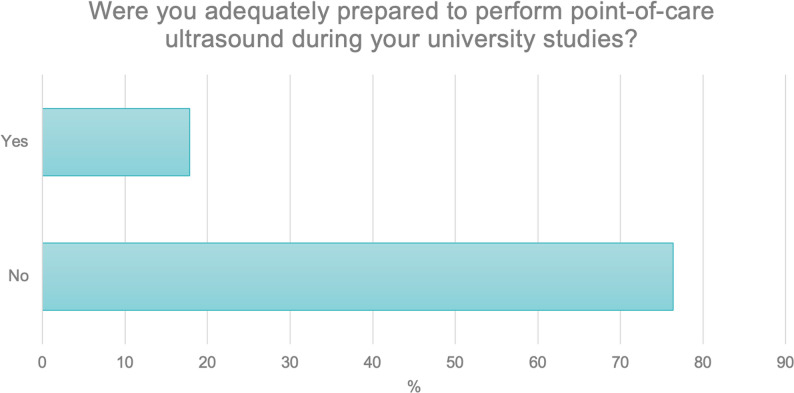
Adequacy of preparation for performing point-of-care ultrasound (POCUS) during university studies

The majority, as many as 76.4% (*N* = 382), indicated that they had not been prepared for such procedures. A total of 17.8% (*N* = 89) reported having received adequate preparation, while 5.6% (*N* = 28) were unsure whether they had undergone such training (Fig. 2).

Respondents were then asked in a multiple-choice question when they would have preferred to be taught the use of point-of-care ultrasound.

**Fig. 3:**
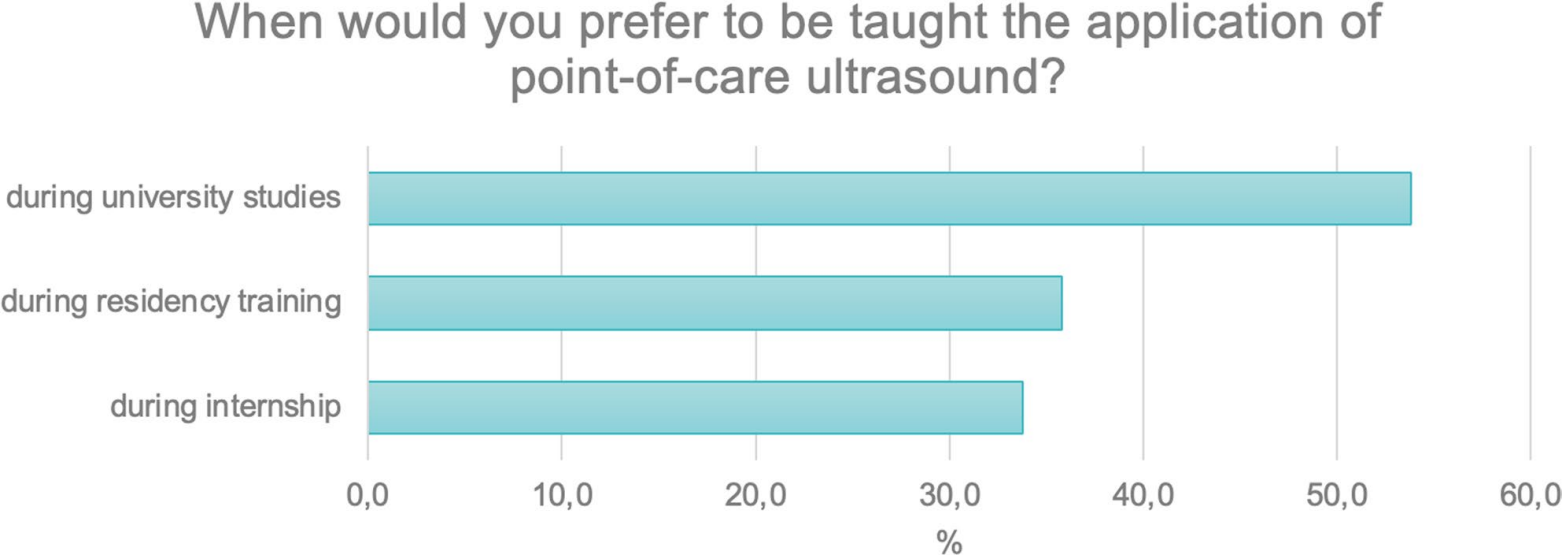
Preferred timing for POCUS training as indicated by respondents

The largest proportion (*N* = 269; 53.8%) indicated that teaching during their university studies would have been preferred. Approximately 30% identified the internship period (*N* = 169; 33,8%) or during residency training (*N* = 179; 35.8%) as the most appropriate time (Fig. 3).

The next question concerned the frequency with which respondents required ultrasound examinations in their clinical practice.

**Fig. 4:**
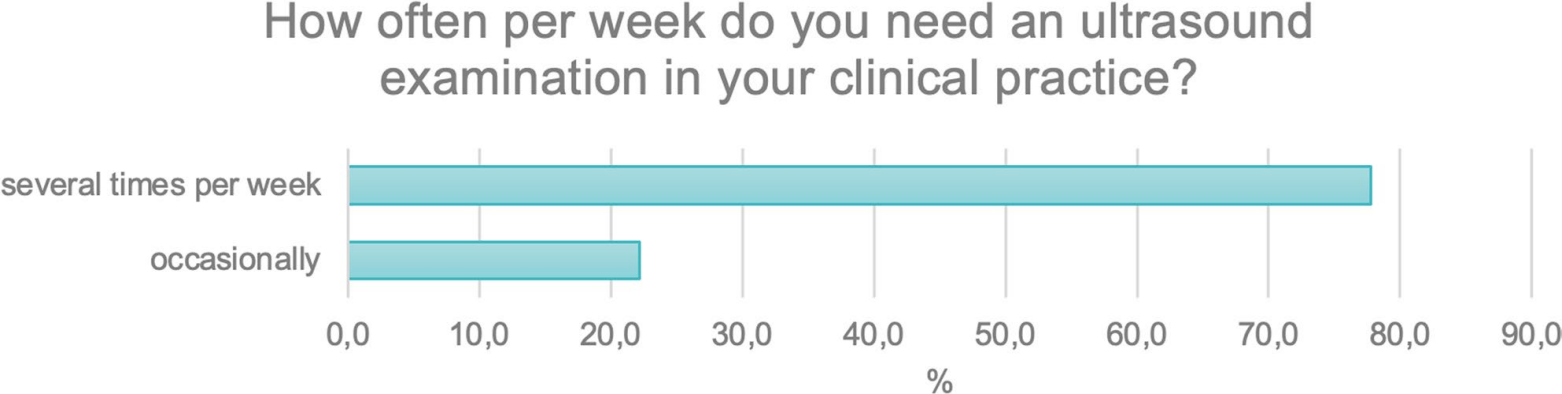
Frequency of ultrasound use in clinical practice on a weekly basis

The analysis revealed that more than 75% of participants (*N* = 389; 77.8%) reported needing ultrasound examinations several times per week, whereas only 22% (*N* = 111; 22.2%) indicated that they required such examinations only occasionally (Fig. 4).

The fifth question focused on respondents’ opinions regarding whether possessing basic ultrasound examination skills improved their clinical work.

**Fig. 5:**
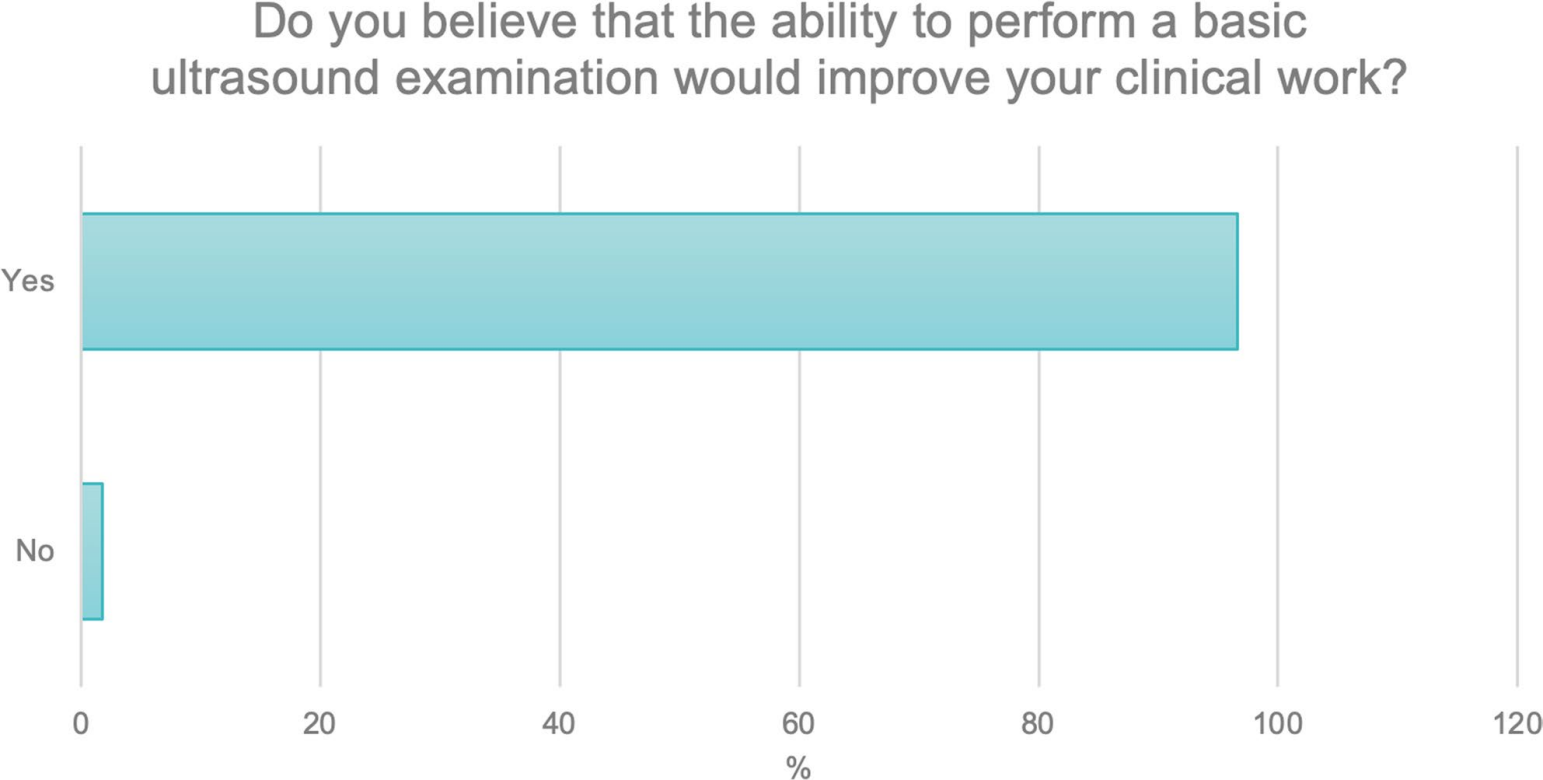
Respondents’ opinions on the impact of basic ultrasound skills on clinical practice

Almost all participants (*N* = 498; 96.2%) provided a positive response. A small proportion, 1.6%, selected “I don’t know,” while 1.8% gave a negative answer (Fig. 5).

**Fig. 6:**
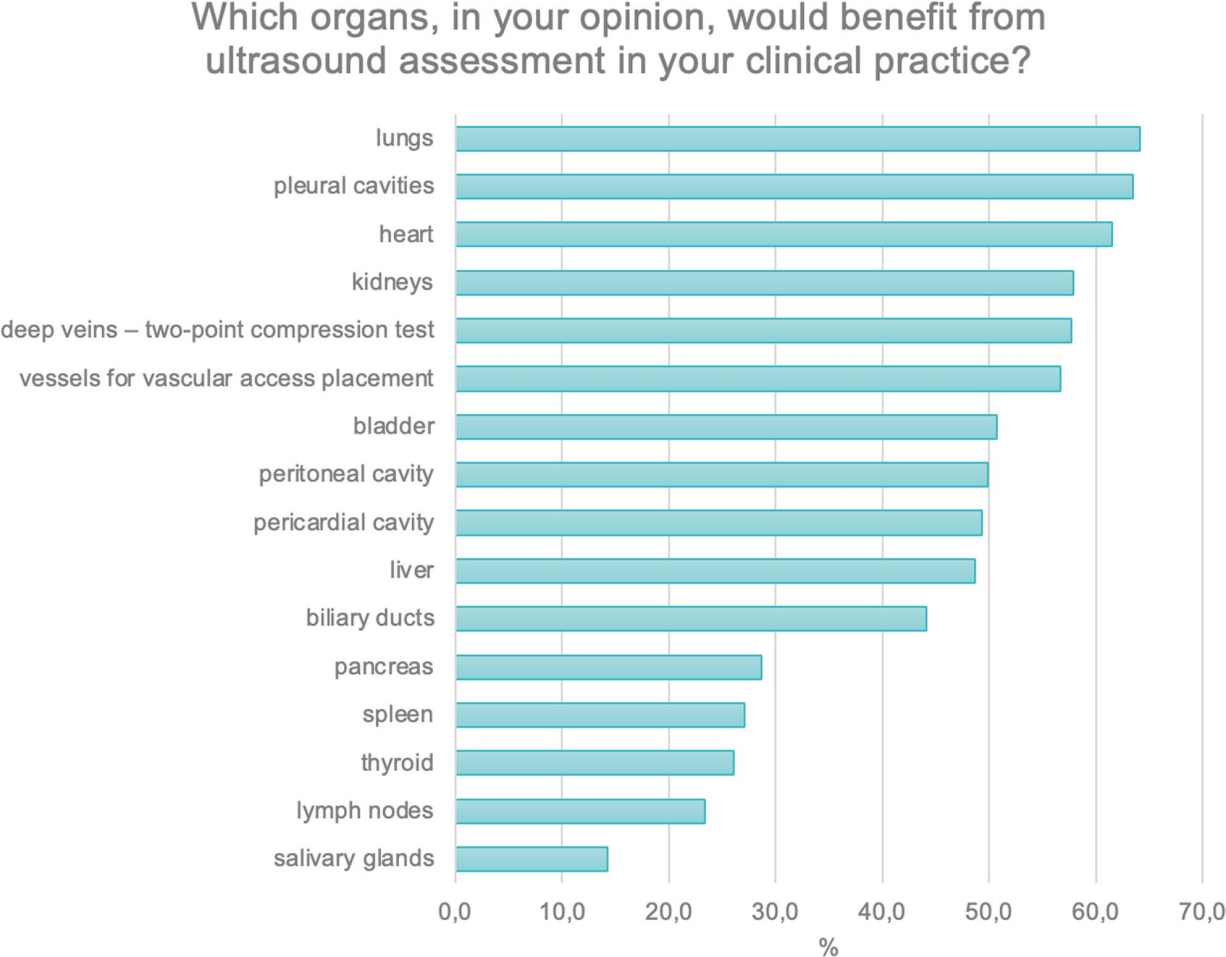
Organs for which ultrasound skills are perceived as useful in daily clinical practice

Another multiple-choice question concerned the organs for which ultrasound examination could be useful in clinical practice. Over 80% of respondents indicated organs located in the chest (*N* = 414; 82.8%), followed by superficially located organs (*N* = 408; 81.6%). The fewest physicians selected abdominal organs (*N* = 396; 79.2%) (Fig. 6).

**Fig. 7:**
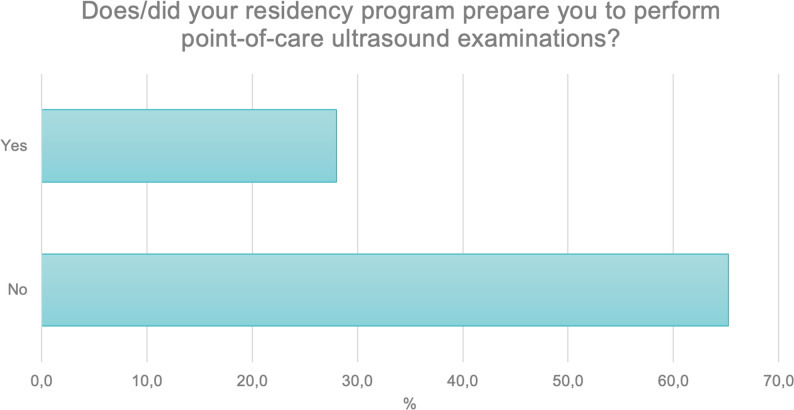
Proportion of respondents who received Point-of-Care Ultrasound (POCUS) training as part of their specialty training program

Participants were then asked whether their specialty training program prepared them to perform point-of-care ultrasound examinations. A total of 65.2% (N = 326) of respondents indicated that their specialty training did not include instruction on this type of examination. A positive response was given by 28% (N = 140), while 6.8% (N = 34) responded “I don’t know (Fig. 7).

**Fig. 8:**
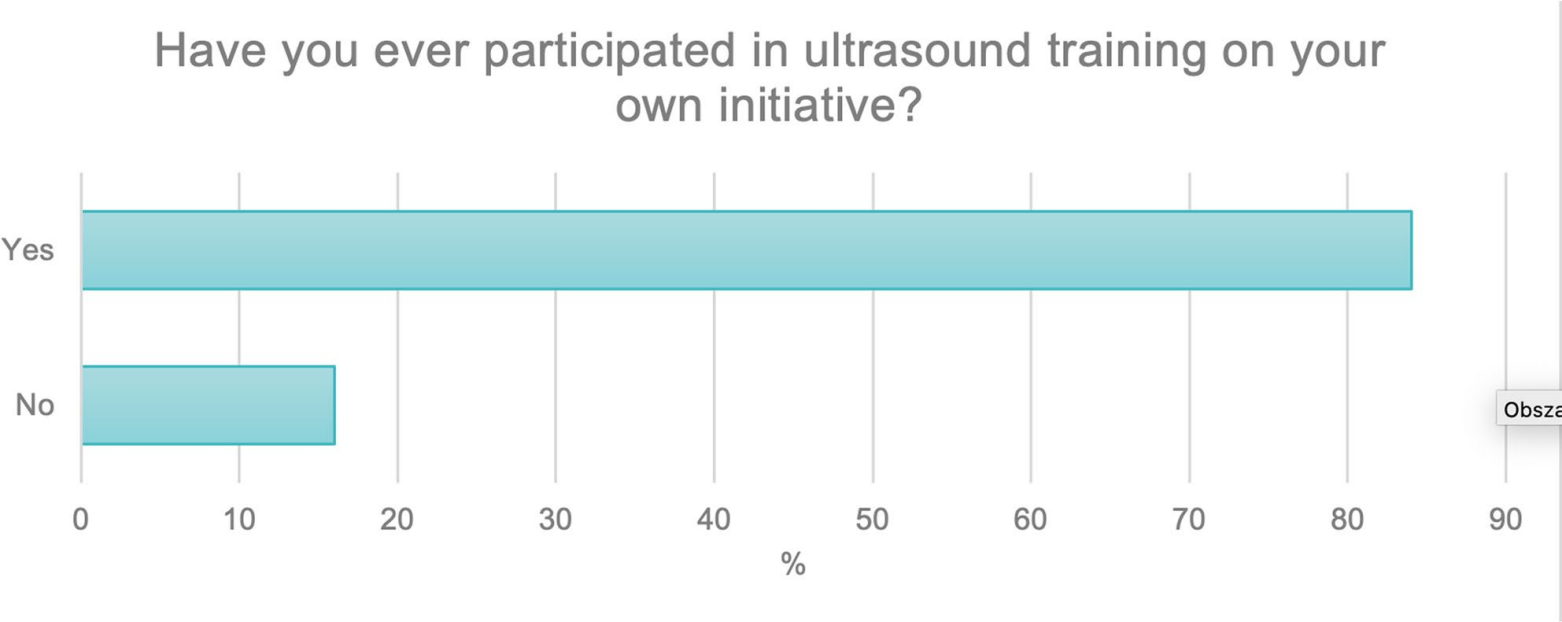
Participation of respondents in external ultrasound courses outside formal undergraduate and postgraduate education

The next question asked participants whether they had engaged in ultrasound training on their own initiative, outside of the national education system at university and beyond their specialty training. More than half of the respondents (*N* = 420; 84%) indicated that they had participated in such training, while 15.8% (*N* = 80) reported that they had not (Fig. 8).

**Fig. 9:**
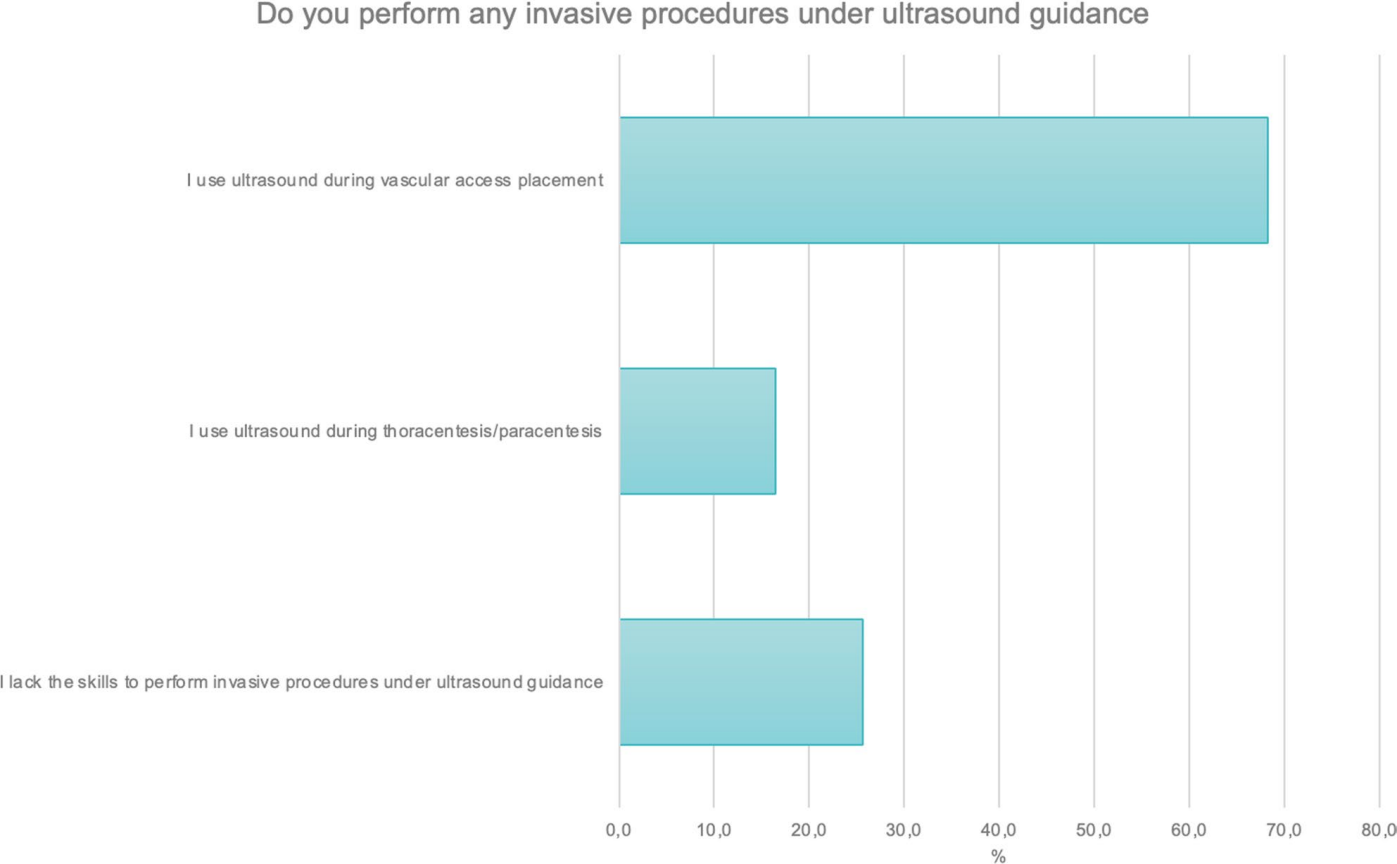
Types of interventional procedures performed by respondents under ultrasound guidance

The survey then asked about interventional procedures performed by respondents under ultrasound guidance. Among the listed methods in this multiple choice question, the highest proportion (*N* = 341; 68.2%) reported using ultrasound to obtain vascular access. Additionally, 1.6% (*N* = 82) used ultrasound during thoracentesis or paracentesis procedures. A total of 25.6% (*N* = 128) indicated that they did not possess the skills to perform interventional procedures under ultrasound guidance (Fig. 9).

**Fig. 10:**
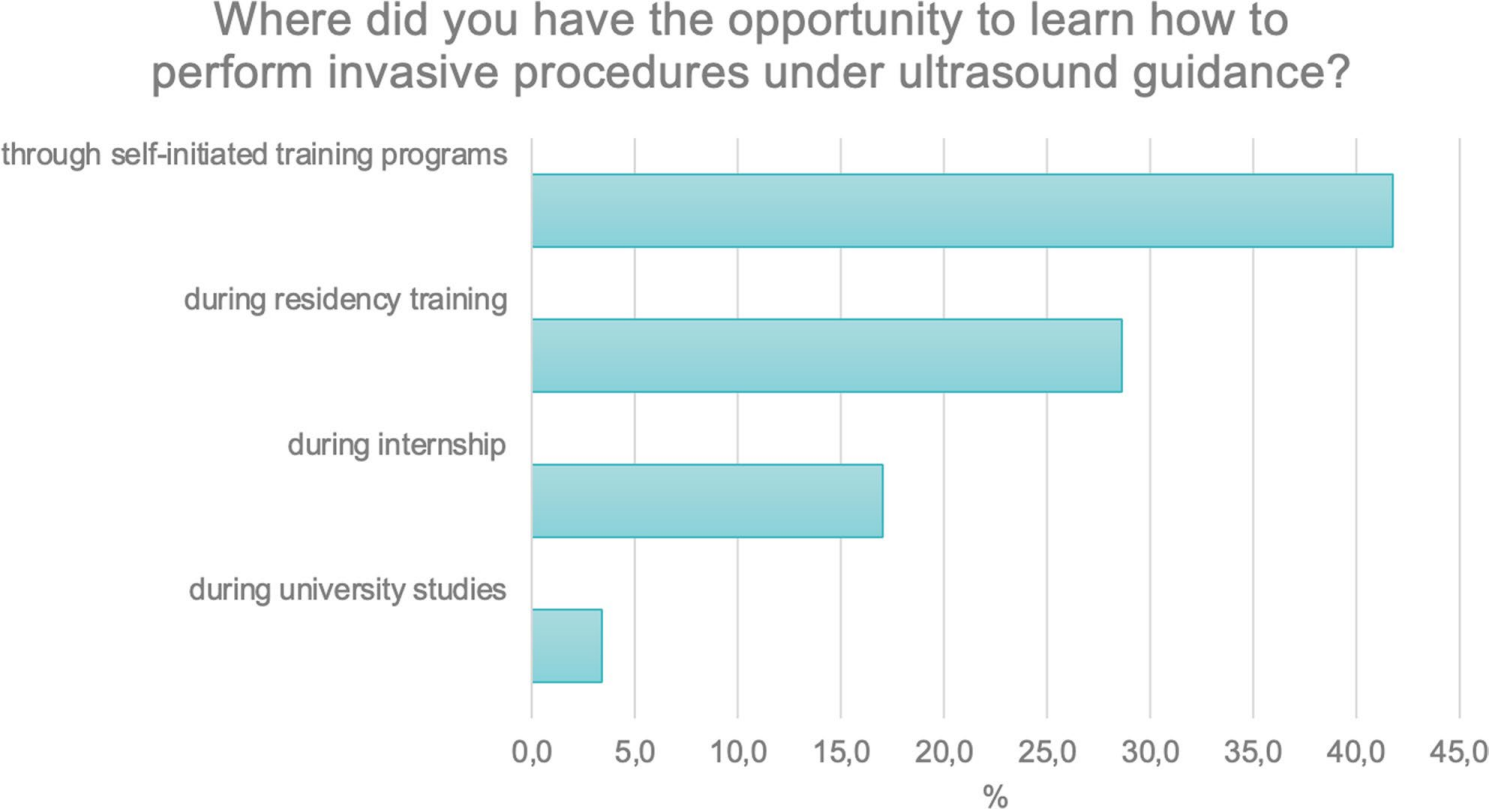
Places where respondents acquired skills in performing ultrasound-guided interventional procedures

The final multiple-choice question concerned where respondents had the opportunity to learn how to perform interventional procedures under ultrasound guidance. The majority of respondents — 41.8% (*N* = 209) — indicated that they acquired these skills through independently organized training. A total of 28.6% (*N* = 143) reported gaining such skills as part of their specialization program, while 17% (*N* = 85) acquired them during their postgraduate internship (Fig. 10).

Notably, the results revealed that interventional procedures under ultrasound guidance were not systematically taught during medical studies or residency training, highlighting a critical gap in current educational programs.

A descriptive analysis by specialty was performed to identify potential differences in ultrasound education and practice among physicians from various disciplines. Emergency medicine physicians most frequently reported regular use of point-of-care ultrasound (POCUS) in daily clinical practice and demonstrated the highest level of confidence interventional procedures. Anesthesiologists emphasized the use of ultrasound primarily for vascular access and imaging structures like heart, lungs, pericardial and pleural cavities. Although point-of-care ultrasound (POCUS) is widely applied in daily clinical practice, only a small proportion of respondents reported that their residency program had adequately prepared them for its use. Internal medicine doctors most often used ultrasound for abdominal and thoracic organ evaluation. However, this group showed the lowest rate of ultrasound education during residency, with the majority acquiring skills through external workshops. Family medicine physicians expressed the greatest interest in learning POCUS at the undergraduate level, highlighting its relevance for primary care settings. Nonetheless, only a few respondents reported having the opportunity to receive formal training in this area during their education and most gained their first experience with ultrasound independently while working in outpatient settings. Overall, the specialty-based analysis revealed that formal ultrasound training remains inconsistent across disciplines, with most participants gaining practical skills independently through self-organized or external courses.

## Discussion

The topic of Point-of-Care Ultrasound (POCUS) education has been widely debated for many years among various organizations and societies involved in ultrasound education. Over the past decade, several publications have outlined key recommendations for teaching POCUS. One notable contribution is the set of guidelines developed by experts at the Seguin Canadian POCUS Conference, resulting in 14 recommendations detailing the optimal structure of POCUS education. The proposals concerned methods of ultrasound education, highlighted potential challenges that may arise during the teaching process, and suggested new solutions that could facilitate didactic efforts. Another significant document is the publication by the World Federation for Ultrasound in Medicine and Biology (WFUMB), which largely aligns with the Canadian guidelines.

A key principle emphasized by both organizations is that POCUS training should begin as early as possible and continue throughout medical education. A single exposure without repeated practice and reinforcement is unlikely to yield lasting proficiency. This finding contrasts with the results of a survey, which indicated that many physicians first handled an ultrasound probe during their regular work in the hospital. However, the majority expressed a desire to have learned POCUS during medical school. Further recommendations highlight that students should be comfortable operating ultrasound probes and interpreting images under minimal supervision, without the immediate expectation of becoming experts. Institutions should offer additional, specialized training opportunities for students particularly interested in POCUS. The curriculum should initially focus on normal physiological imaging, with the introduction of pathological findings only as students advance. Such an approach enables students to recognize abnormalities based on a solid understanding of normal anatomy [11, 12].

WFUMB additionally emphasized the importance of teaching basic ultrasound machine operation—often referred to as “knobology”—which serves as the foundation for image optimization and the acquisition of correct ultrasound views [13]. Another essential element is thorough training in ultrasound scanning techniques, ensuring students are prepared to perform scans in diverse settings, such as primary care offices and resuscitation rooms.

Terminology standardization was also proposed to enhance communication, both between students and instructors and later in clinical practice. WFUMB underscored the superiority of modern educational methods, like online platforms and self-directed learning, over traditional lectures and seminars. Although lectures allow for the training of large groups without the need for new methodologies, they often decrease student motivation and consume time that could be better spent practicing practical skills. Notably, only 4% of survey respondents reported exposure to such modern teaching methods [10, 14].

An example of an effective POCUS educational system is the University of South Carolina School of Medicine, which has offered a comprehensive four-year ultrasound curriculum since 2006. Each year of the program is structured systematically, requiring students to pass OSCE (Objective Structured Clinical Examination) assessments at the end of each phase to advance. Early years emphasize theoretical subjects, such as sonoanatomy, equipment operation, and ultrasound safety. As students progress, the curriculum increasingly focuses on bedside ultrasound practice across various clinical settings, including internal medicine, intensive care, and emergency departments. The program utilizes diverse teaching methods, including small group clinical sessions, near-peer teaching, online platforms for video review and expert feedback, and simulator-based training. In contrast, lectures and seminars form only a minor part of the syllabus, unlike traditional medical education programs in Poland [15].

In the Polish medical education system, ultrasound teaching remains largely optional and inconsistent. Integrating POCUS elements into preclinical courses - particularly anatomy, physiology, and the introduction to internal medicine, could greatly enhance spatial understanding and facilitate the transition from theoretical knowledge to clinical application. The inclusion of short, hands-on ultrasound modules during the early years of study, using portable ultrasound devices, would allow students to gradually build confidence in image acquisition and interpretation.

Another potential improvement would be the development of a national framework for ultrasound education, modeled on WFUMB and European recommendations. Such a framework could include basic theoretical training (covering ultrasound physics, anatomy, and knobology) combined with structured clinical workshops. The introduction of near-peer teaching—where senior students train junior ones under instructor supervision—could help overcome the shortage of qualified tutors and enhance the learning process.

Finally, strengthening simulation infrastructure and ensuring broader access to portable ultrasound devices at medical universities should become a national priority. The use of high-fidelity simulators, anatomical phantoms, and e-learning platforms with access to real clinical cases could create an integrated, modern learning environment. This approach, based on active learning and practical skill assessment (e.g., OSCE), would contribute to the standardization of ultrasound competency levels among graduates of Polish medical schools within the coming years.

Standardizing POCUS education across medical schools, internships, and residency programs would offer significant benefits. Notably, all physicians, regardless of specialty, would possess consistent ultrasound skills, enabling rapid visualization and verification of clinical findings obtained through history taking, physical examination, or monitoring patient parameters [1]. A unified educational approach would also standardize terminology and communication, facilitating collaboration during training and clinical practice [10].

Our findings confirm that ultrasound-guided interventional procedures are increasingly used in clinical practice, yet structured education in this field remains limited. More than two-thirds of respondents reported performing ultrasound-guided vascular access, while only a small proportion had received formal training in interventional techniques. This suggests that many physicians acquire these competencies independently, outside formal educational programs. This observation is consistent with previous studies emphasizing that ultrasound guidance has become a standard of care in procedures such as central venous catheterization, thoracentesis, and paracentesis, but training opportunities are still heterogeneous and often self-directed [16, 17]. According to the WFUMB position statement, interventional ultrasound skills should be introduced early in postgraduate education and standardized across specialties to ensure patient safety and procedural efficacy [10]. Similarly, Saugel et al. have shown that structured ultrasound-guided training improves procedural success rates and significantly reduces complications [18, 19]. Therefore, the results of our survey highlight the urgent need to incorporate hands-on training in ultrasound-guided interventions into both residency and continuing professional education. Establishing standardized curricula and competency-based assessments would help bridge the gap between clinical demand and current training availability across Europe.

The absence of standardized ultrasound education in the four surveyed European countries highlights a major gap in medical training. Although point-of-care ultrasound (POCUS) is increasingly recognized as an essential diagnostic and procedural skill, its integration into undergraduate and postgraduate curricula remains inconsistent. In most participating countries, ultrasound training is either optional, limited to radiology courses, or offered only through external workshops. Consequently, the acquisition of practical ultrasound skills often depends on individual initiative rather than structured educational pathways. This lack of formal training leads to unequal access to ultrasound education and contributes to significant variability in the competencies of practicing physicians. Our results reflect this disparity, as the majority of respondents indicated that they had to pursue additional courses independently to acquire POCUS skills. These findings are consistent with the WFUMB position paper, which emphasizes that ultrasound should be introduced as a core element of medical education and systematically reinforced during postgraduate training [10]. Addressing this educational gap through unified European curricula would ensure equitable skill development and ultimately improve the quality and safety of patient care.

At the beginning of the 19th century, the introduction of the stethoscope faced considerable skepticism, with critics calling it impractical and inconvenient [20]. Yet, the stethoscope eventually became indispensable for physicians, nurses, and paramedics alike. Similarly, today, ultrasound has become a crucial diagnostic tool. Thanks to increasing functionality, portability, and affordability, modern ultrasound devices allow clinicians to supplement and verify findings from history taking and physical examination at the patient’s bedside [1, 3, 4]. However, achieving high and consistent proficiency in POCUS requires a fundamental modification of current education systems at both the undergraduate and postgraduate levels.

### Limitations

This study has several limitations that should be acknowledged.

First, the survey was conducted exclusively among physicians from four European countries (Poland, Austria, Spain, and Portugal). Since educational systems and approaches to ultrasound teaching differ considerably across regions, the findings may not be fully generalizable to other continents such as North America or Asia.

Second, although both residents and specialists participated in the study, the questionnaire did not record the exact time since graduation or the country in which respondents completed their medical education. As a result, potential differences between earlier and more recently trained physicians, as well as between those educated in different national systems, could not be analyzed.

Third, the survey included physicians from various specialties, however, some groups, particularly those with lower ultrasound use (e.g., neurology, sports medicine), were underrepresented. Therefore, the results mainly reflect the perspectives of clinicians in ultrasound-intensive fields such as internal medicine, emergency medicine, anesthesiology, and family medicine.

Fourth, subgroup analyses by country and specialty were descriptive in nature, and the data were self-reported. This introduces potential recall and response bias, which is inherent to survey-based research.

Finally, the study design did not include validation of respondents’ self-assessed ultrasound skills through objective testing. Future studies should combine questionnaire data with practical performance assessments to provide a more comprehensive evaluation of ultrasound competencies among physicians.

This study also has several notable strengths.

First, it represents one of the few multicountry surveys evaluating Point-of-Care Ultrasound (POCUS) education among practicing physicians in Europe. The inclusion of participants from four countries (Poland, Austria, Spain, and Portugal) provides a valuable international perspective on current educational practices and unmet needs.

Moreover, the study sample was relatively large (*N* = 500) and encompassed physicians from a wide range of specialties, including internal medicine, anesthesiology, surgery, emergency medicine, and pediatrics. This diversity allowed for a comprehensive overview of ultrasound training experiences across different clinical contexts.

Furthermore, the questionnaire addressed not only current proficiency and training history but also participants’ expectations regarding the optimal timing of POCUS education. This combination of retrospective and prospective viewpoints offers important guidance for future curriculum development.

Lastly, the study’s findings highlight the strong, consistent demand for earlier and structured ultrasound education in medical training programs, reinforcing existing international recommendations and providing evidence that can inform future educational policy in Europe.

## Conclusion

Survey responses reveal a strong demand for POCUS education beginning at the undergraduate and postgraduate internship levels. The vast majority of physicians did not acquire POCUS skills during their medical education or specialty training. However, most respondents agreed that ultrasound skills would significantly enhance their clinical practice. There is a clear need to standardize and uniformly implement POCUS education across all medical schools offering healthcare education programs.

## Supplementary Information

Supplementary Material 1.

Supplementary Material 2.

## Data Availability

The datasets generated and analyzed during the current study are available from the corresponding author on reasonable request.
