# Supplementary material for: POCUS teaching - needs and reality based on 500 respondents
Source: BMC Med Educ. 2026 Feb 25;26:530. doi: 10.1186/s12909-026-08860-1 (PMC13041445; doi:10.1186/s12909-026-08860-1)
Supplement: Supplementary file 1 — Supplementary Material 1. [file 12909_2026_8860_MOESM1_ESM.docx]

**Question 1 – multiple choice**

*When did you first have the opportunity to perform an ultrasound examination independently?*

1. Never
2. During regular coursework at university
3. During extracurricular activities at university
4. During residency training
5. While working in a hospital
6. While working in an outpatient clinic

**Question 2**

*Were you adequately prepared to perform point-of-care ultrasound during your university studies?*

1. No
2. I don’t know
3. Yes

**Question 3 - multiple-choice**

*When would you have preferred to be taught the use of point-of-care ultrasound?*

1. During internship
2. During residency training
3. During university studies

**Question 4**

*How often do you require ultrasound examinations in your clinical practice on a weekly basis?*

1. Occasionally
2. Several times per week

**Question 5**

*In your opinion, would having the ability to perform a basic ultrasound examination improve* *your clinical practice?*

1. No
2. I don’t know
3. Yes

**Question 6 - multiple-choice**

*Which organs' ultrasound assessment skills could be useful in your clinical practice?*

1. Lungs
2. Pleural cavities
3. Heart
4. Pericardial cavity
5. Peritoneal cavity
6. Kidneys
7. Bladder
8. Liver
9. Biliary ducts
10. Pancreas
11. Spleen
12. Thyroid
13. Salivary glands
14. Lymph nodes
15. Deep veins – two-point compression test
16. Vessels for vascular access placement

**Question 7**

*Does/did your specialty training program prepare you to perform point-of-care ultrasound examinations?*

1. No
2. I don’t know
3. Yes

**Question 8**

*Have you ever participated in ultrasound training on your own initiative, outside of the national education system at university and beyond your specialty training?*

1. Yes
2. No

**Question 9 - multiple-choice**

*Which interventional procedures do you perform under ultrasound guidance?*

1. I lack the skills to perform invasive procedures under ultrasound guidance
2. I use ultrasound during thoracentesis/paracentesis
3. I use ultrasound during vascular access placement

**Question 10 - multiple-choice**

*Where do you have the opportunity to learn how to perform interventional procedures under ultrasound guidance?*

1. During university studies
2. During internship
3. During residency training
4. During self-initiated training programs
