# Supplementary material for: POCUS teaching - needs and reality based on 500 respondents
Source: BMC Med Educ. 2026 Feb 25;26:530. doi: 10.1186/s12909-026-08860-1 (PMC13041445; doi:10.1186/s12909-026-08860-1)
Supplement: Supplementary file 2 — Supplementary Material 2. [file 12909_2026_8860_MOESM2_ESM.docx]

**Characteristics of responses – Austria (N = 70)**

The first survey question concerned the moment when respondents first had the opportunity to hold an ultrasound probe and attempt to perform an examination.
A total of **34.3% (N = 24)** reported that this occurred during **regular university classes**, while **28.6% (N = 20)** indicated that it happened **during extracurricular activities**.
For **25.7% (N = 18)** of participants, this experience took place **during their internship**, and **7.1% (N = 5)** reported that it occurred **during residency training**.
One respondent each (1.4%) stated that this happened **while working in a hospital or outpatient clinic**, or that they **had never held an ultrasound probe before**.

**Figure 1.**
Percentage distribution of responses to the question: “When did you first have the opportunity to hold an ultrasound probe and attempt to perform an examination?”

In the next question, respondents were asked about their preparation for performing point-of-care ultrasound (POCUS) during their university studies. The majority of participants — 82.9% (N = 58) — indicated that they had not been prepared to perform such examinations.
A total of 12.9% (N = 9) reported that they had received adequate preparation, while 4.3% (N = 3) were unsure whether such training had been included in their studies (selected the response *“I don’t know”*).

**Figure 2.**
Percentage distribution of responses to the question: *“Were you adequately prepared to perform point-of-care ultrasound (POCUS) during your university studies?”*

Respondents were then asked when they would prefer to be taught the application of point-of-care ultrasound (POCUS). The majority (N = 44; 62.9%) indicated during medical school. Thirty percent (N = 21) selected the internship period, and only five respondents (7.1%) indicated residency training.

**Figure 3**
Percentage distribution of responses to the question: *When would you prefer to be taught the application of point-of-care ultrasound?*

The next question concerned the frequency with which respondents require ultrasound examinations in their clinical practice. The analysis showed that over 40% (N = 31; 44.3%) reported needing such examinations almost daily, 32.9% (N = 23) several times per week, 14.3% (N = 10) once per week, and 8.6% (N = 6) only occasionally.

**Figure 4**
Percentage distribution of responses to the question: *How often per week do you need an ultrasound examination in your clinical practice?*

The next question addressed respondents’ opinions on whether having the ability to perform a basic ultrasound examination would improve their clinical work. Nearly all participants (N = 68; 87.1%) responded positively. Only two individuals (2.9%) gave a negative response.

**Figure 5**

*Percentage distribution of responses to the question: Do you believe that the ability to perform a basic ultrasound examination would improve your clinical work?*

The following question asked respondents to indicate which organs they believed would benefit from ultrasound assessment in their clinical practice. Over 80% identified the kidneys (N = 58; 82.9%) and the heart (N = 57; 81.4%) as most relevant. Approximately 60% of respondents selected the pleural cavities (N = 46; 65.7%), lungs (N = 43; 61.4%), and deep veins (N=41;58.6%). Additionally, 54.3% (N = 38) indicated the bladder, and 53.9% (N = 37) the pericardial sac. Around 40% found ultrasound useful for assessing the liver (N = 32; 45.7%), vascular access placement (N = 31; 44.3%), the peritoneal cavity (N = 29; 41.4%), and the biliary ducts (N = 27; 38.6%). The spleen was selected by 24.3% (N = 17), while between 10–15% of respondents indicated the pancreas (N = 11; 15.7%), thyroid gland (N = 10; 14.3%), and lymph nodes (N = 8; 11.4%).
Only 4.3% (N = 3) considered ultrasound assessment of the salivary glands to be clinically useful.

**Figure 6**
Percentage distribution of responses to the question: *Which organs, in your opinion, would benefit from ultrasound assessment in your clinical practice?*

Respondents were then asked whether their residency program prepares or has prepared them to perform point-of-care ultrasound (POCUS) examinations. Over half of the respondents (N = 37; 52.9%) answered negatively. A total of 40% (N = 28) indicated that they had received such preparation, while 7.1% (N = 5) responded “I don't know.”

**Figure 7**
Percentage distribution of responses to the question: *Does/did your residency program prepare you to perform point-of-care ultrasound examinations?*

Respondents were then asked whether they had participated in ultrasound training courses on their own initiative. Nearly all had done so—either multiple times (N = 50; 71.4%) or occasionally (N = 17; 24.3%). Only 3 individuals (4.3%) reported never having attended such training.

**Figure 8**
Percentage distribution of responses to the question: *Have you ever participated in ultrasound training on your own initiative?*

Respondents were then asked about invasive procedures they perform under ultrasound guidance. Among the listed procedures, the highest proportion (N = 35; 50.0%) reported using ultrasound for vascular access placement. Additionally, 28.6% (N = 20) used ultrasound during thoracentesis or paracentesis. A total of 21.4% (N = 15) indicated that they do not possess the skills required to perform such procedures.

**Figure 9**
Percentage distribution of responses to the question: *Do you perform any invasive procedures under ultrasound guidance?*

The final question asked respondents where they had the opportunity to learn how to perform invasive procedures under ultrasound guidance. The majority (41.4%; N = 29) reported acquiring these skills during residency training. A total of 28.6% (N = 20) gained such experience during their internship, while 27.1% (N = 19) participated in self-initiated training courses. Only two respondents (2.9%) indicated that they had such opportunities during medical school.

**Figure 10.**
Percentage distribution of responses to the question: *Where did you have the opportunity to learn how to perform invasive procedures under ultrasound guidance?*

**Characteristics of Responses – Spain (N = 218)**

The first question asked respondents when they first had the opportunity to hold an ultrasound probe and attempt an ultrasound examination. Approximately 20% of participants reported gaining this experience during residency training (N = 53; 24.3%), while 21.1% (N = 46) indicated that it occurred while working in a hospital, and 20.2% (N = 44) during extracurricular activities at university. A total of 19.7% (N = 43) reported performing ultrasound examinations while working in an outpatient clinic. Additionally, 7.8% (N = 17) indicated the internship period (N = 46; 9.2%), and only 2.8% (N = 6) reported performing ultrasound as part of regular university coursework.
Finally, 4.1% (N = 9) of respondents stated that they had no prior experience with ultrasound.

**Figure 11.**Percentage distribution of responses to the question: *When did you first have the opportunity to hold an ultrasound probe and attempt an ultrasound examination?*

Respondents were then asked whether they had received any training in performing point-of-care ultrasound (POCUS) during their medical studies. The majority of participants (66.5%; N = 145) reported that they had not received such preparation. A total of 23.9% (N = 52) indicated that they had undergone such training, while 9.2% (N = 20) were unsure whether they had received it.

**Figure 12**
Percentage distribution of responses to the question: *Were you trained to perform point-of-care ultrasound during your medical studies?*

Respondents were then asked when they would prefer to be taught the application of point-of-care ultrasound (POCUS). The largest proportion (N = 91; 41.7%) indicated during medical school, while a slightly smaller group (38.1%; N = 83) selected residency training. A total of 19.3% (N = 42) stated that they would prefer to acquire these skills during their internship.

**Figure 13**
Percentage distribution of responses to the question: *When would you prefer to be taught the application of point-of-care ultrasound?*

The next question concerned how often respondents need to perform ultrasound examinations in their clinical work. Nearly half of the participants (N = 105; 48.2%) reported requiring such examinations almost daily, 28.9% (N = 63) several times per week, 14.7% (N = 32) once per week, and 8.3% (N = 18) stated that they need such examinations only occasionally.

**Figure 14**
Percentage distribution of responses to the question: *How often per week do you need an ultrasound examination in your clinical practice?*

The next question addressed respondents’ opinions on whether having the ability to perform a basic ultrasound examination would improve their clinical work. Nearly all participants (N = 205; 94.0%) responded positively. A total of 1.8% (N = 4) selected the response “I don’t know,” while 3.7% (N = 8) gave a negative answer.

**Figure 15**

*Percentage distribution of responses to the question: Do you believe that the ability to perform a basic ultrasound examination would improve your clinical work?*

The next question asked respondents to indicate which organs they believed would benefit from ultrasound assessment in their clinical practice. More than half of the participants identified the lungs (N = 132; 60.8%), kidneys (N = 131; 60.4%), liver (N = 129; 59.4%), biliary ducts (N = 124; 57.1%), deep veins (N = 122; 56.2%), and heart (N = 117; 53.9%) as the most relevant. A slightly smaller proportion—over 40%—indicated the pleural cavities (N = 108; 49.8%), bladder (N = 103; 47.5%), spleen (N = 102; 47.0%), pericardial sac (N = 94; 43.3%), and peritoneal cavity (N = 90; 41.5%). More than 30% of respondents stated that ultrasound would be useful for assessing the pancreas (N = 85; 39.2%), vessels for vascular access placement (N = 83; 38.2%), and thyroid gland (N = 77; 35.5%). Additionally, 28.6% (N = 62) selected lymph nodes, and 21.2% (N = 46) indicated salivary glands as organs for which ultrasound could be beneficial in their work.

**Figure 16**
Percentage distribution of responses to the question: *Which organs, in your opinion, would benefit from ultrasound assessment in your clinical practice?*

Respondents were then asked whether their residency program prepares or has prepared them to perform point-of-care ultrasound (POCUS) examinations. A total of 61.5% (N = 134) stated that their residency program does not include training in this type of examination. In contrast, 35.8% (N = 78) responded affirmatively, while 2.8% (N = 6) selected the response “I don’t know.”

**Figure 17**
Percentage distribution of responses to the question: *Does/did your residency program prepare you to perform point-of-care ultrasound examinations?*

Respondents were then asked whether they had participated in ultrasound training courses on their own initiative. More than 80% reported having done so—either occasionally (N = 45; 20.6%) or multiple times (N = 138; 63.3%). A total of 15.6% (N = 34) indicated that they had never taken part in such self-initiated training.

**Figure 18**
Percentage distribution of responses to the question: *Have you ever participated in ultrasound training on your own initiative?*

Respondents were next asked about invasive procedures they perform under ultrasound guidance. The analysis showed that the largest proportion (N = 82; 37.6%) reported using ultrasound during thoracentesis or paracentesis, while 25.2% (N = 55) used ultrasound for vascular access placement. A total of 36.2% (N = 79) indicated that they do not possess the skills required to perform invasive procedures under ultrasound guidance.

**Figure 19**
Percentage distribution of responses to the question: *Do you perform any invasive procedures under ultrasound guidance?*

The final question concerned where respondents had the opportunity to learn how to perform invasive procedures under ultrasound guidance. The majority (45.9%; N = 100) reported acquiring these skills through self-initiated training courses. A total of 32.1% (N = 70) gained such experience during residency training, 16.5% (N = 36) during their internship, and only 5% (N = 11) during medical school.

**Figure 20**
Percentage distribution of responses to the question: *Where did you have the opportunity to learn how to perform invasive procedures under ultrasound guidance?*

**Characteristics of Responses – Poland (N = 127)**

The first survey question asked respondents when they first had the opportunity to hold an ultrasound probe and attempt an ultrasound examination. Nearly 30% of participants indicated that this experience occurred during extracurricular activities at university (N = 37; 29.1%). A total of 25.2% (N = 32) reported gaining such experience during residency training, and 22.8% (N = 29) while working in a hospital. Furthermore, 11.8% (N = 15) first held an ultrasound probe during their internship, and 7.9% (N = 10) during regular university coursework. A total of 2.4% (N = 3) reported no prior experience, while one respondent (0.8%) indicated gaining this experience while working in an outpatient clinic.

**Figure 21**
Percentage distribution of responses to the question: “When did you first have the opportunity to hold an ultrasound probe and attempt to perform an examination?”

Respondents were then asked whether they had been prepared during their medical studies to perform point-of-care ultrasound (POCUS) examinations. Almost all participants—91.3% (N = 116)—reported that they had not received such preparation. A total of 7.1% (N = 9) indicated that they had received relevant training, while 1.6% (N = 2) were unsure whether they had undergone such preparation.

**Figure 22**
Percentage distribution of responses to the question: *“Were you adequately prepared to perform point-o2f-care ultrasound (POCUS) during your university studies?”*

Respondents were then asked at which stage of their education they would prefer to be taught the application of point-of-care ultrasound (POCUS). The largest proportion (N = 208; 43.3%) indicated multiple stages—medical school, internship, and residency training. Thirty-seven percent (N = 47) stated that they would like to acquire these skills during medical school. Fewer than 10% selected residency training (N = 11; 8.7%), while less than 5% indicated internship (N = 5; 3.9%), both internship and medical school (N = 5; 3.9%), internship and residency (N = 3; 2.4%), and one respondent (0.8%) selected medical school and residency.

**Figure 23**
Percentage distribution of responses to the question: *When would you prefer to be taught the application of point-of-care ultrasound?*

The fourth question concerned how often respondents require ultrasound examinations in their clinical practice. The analysis showed that more than half of the participants (N = 65; 51.2%) reported needing such examinations almost daily, 35.4% (N = 45) several times per week, 10.2% (N = 13) once per week, and 3.1% (N = 4) stated that they need such examinations only occasionally.

**Figure 24**
Percentage distribution of responses to the question: *How often per week do you need an ultrasound examination in your clinical practice?*

The fifth question addressed respondents’ opinions on whether having the ability to perform a basic ultrasound examination would improve their clinical work. All respondents except one (N = 126; 99.2%) answered positively. One participant provided a negative response.

**Figure 225**

*Percentage distribution of responses to the question: Do you believe that the ability to perform a basic ultrasound examination would improve your clinical work?*

The sixth question concerned the perceived usefulness of ultrasound in examining various organs during clinical practice. The majority of respondents indicated that ultrasound is most useful for assessing the pleural cavities (N = 116; 91.3%), vascular access placement (N = 103; 81.1%), and lungs (N = 101; 79.5%). Approximately 70% considered ultrasound useful for examining the pericardial sac (N = 92; 72.4%), heart, deep veins, and peritoneal cavity (each N = 88; 69.3%). A total of 64.6% (N = 82) reported the need for ultrasound when examining the bladder, and 61.4% (N = 78) when examining the kidneys.
Around 40% of participants identified the liver (N = 61; 48.0%) and biliary ducts (N = 52; 40.9%) as organs where ultrasound would be useful. Between 20% and 30% mentioned the pancreas (N = 36; 28.3%), thyroid gland (N = 28; 22.0%), and lymph nodes (N = 32; 25.2%).
The smallest proportion of respondents selected the salivary glands (N = 18; 14.2%), while none indicated the spleen.

**Figure 26**
Percentage distribution of responses to the question: *Which organs, in your opinion, would benefit from ultrasound assessment in your clinical practice?*

Respondents were then asked whether their residency program prepares or has prepared them to perform point-of-care ultrasound (POCUS) examinations. The vast majority (70.9%; N = 90) stated that their program did not provide such preparation. A total of 11.8% (N = 15) responded affirmatively, while 17.3% (N = 22) selected the response “I don’t know.”

**Figure 27**
Percentage distribution of responses to the question: *Does/did your residency program prepare you to perform point-of-care ultrasound examinations?*

Respondents were then asked whether they had participated in ultrasound training courses on their own initiative. Approximately 40% reported attending such courses multiple times (N = 52; 40.9%) or occasionally (N = 49; 38.6%). A total of 20.5% (N = 26) stated that they had never participated in any self-initiated ultrasound training.

**Figure 28**
Percentage distribution of responses to the question: *Have you ever participated in ultrasound training on your own initiative?*

The ninth question asked respondents about invasive procedures they perform under ultrasound guidance. Over 30% of participants reported using ultrasound both during thoracentesis/paracentesis and for vascular access placement (N = 41; 32.3%). A total of 23.6% (N = 30) used ultrasound exclusively for vascular access placement.
Eleven percent (N = 14) indicated that they lacked the necessary skills and referred such procedures to more experienced colleagues. Fewer than 10% reported delegating these procedures to experienced personnel (N = 12; 9.4%), using ultrasound during thoracentesis/paracentesis or referring the procedure to others (N = 10; 7.9%), or having no skills in this area (N = 8; 6.3%). Only 4.7% (N = 6) of respondents reported using ultrasound solely for thoracentesis or paracentesis.

**Figure 29**
Percentage distribution of responses to the question: *Do you perform any invasive procedures under ultrasound guidance?*

The final question asked respondents where they had the opportunity to learn how to perform invasive procedures under ultrasound guidance. The majority (45.7%; N = 58) reported acquiring these skills through self-initiated training courses. A total of 25.2% (N = 32) indicated gaining such experience through residency training combined with individual courses, while 15.7% (N = 20) acquired these skills exclusively during residency training.
Fewer than 5% reported obtaining such skills through training, residency, and internship combined (N = 6; 4.7%), residency and internship (N = 4; 3.1%), internship alone (N = 3; 2.4%), or both medical school and residency (N = 1; 0.8%). One respondent (0.8%) indicated acquiring these skills through internship, residency, and medical school, and two participants (1.6%) reported having learned them through all of the above pathways.

**Figure 30**
Percentage distribution of responses to the question: *Where did you have the opportunity to learn how to perform invasive procedures under ultrasound guidance?*

**Characteristics of Responses – Portugal (N = 85)**

The first survey question asked respondents when they first had the opportunity to hold an ultrasound probe and attempt an ultrasound examination. A total of 36.5% (N = 31) reported that this occurred during their internship, while 28.2% (N = 24) indicated that it took place while working in a hospital. During residency training, 14.1% (N = 12) of respondents gained this experience, and 7.1% (N = 6) stated that it happened during extracurricular activities at university. A total of 5.9% (N = 5) performed an ultrasound examination for the first time as part of regular university coursework, and one respondent reported performing it for the first time in an outpatient clinic. Additionally, 7.1% (N = 6) indicated that they had never performed an ultrasound examination.

**Figure 31**Percentage distribution of responses to the question: *“When did you first have the opportunity to hold an ultrasound probe and attempt to perform an examination?”*

Respondents were then asked whether they had been prepared during their medical studies to perform point-of-care ultrasound (POCUS) examinations. The majority of participants (74.1%; N = 63) reported that they had not received such preparation. A total of 22.4% (N = 19) indicated that they had undergone relevant training, while 3.5% (N = 3) were unsure whether they had received such preparation.

**Figure 32**
Percentage distribution of responses to the question: *“Were you adequately prepared to perform point-of-care ultrasound (POCUS) during your university studies?”*

Respondents were then asked when they would prefer to be taught the application of point-of-care ultrasound (POCUS). The largest proportion (N = 38; 44.7%) indicated during their internship, while 30.6% (N = 26) selected medical school. A total of 24.7% (N = 21) stated that they would like to acquire these skills during residency training.

**Figure 33**
Percentage distribution of responses to the question: *When would you prefer to be taught the application of point-of-care ultrasound?*

The next question concerned how often respondents require ultrasound examinations in their clinical practice. The analysis showed that more than 40% of participants (N = 39; 45.9%) reported needing such examinations several times per week, 21.2% (N = 18) indicated daily use, 20% (N = 17) reported occasional need, and 12.9% (N = 11) stated that they require such examinations once per week.

**Figure 34**
Percentage distribution of responses to the question: *How often per week do you need an ultrasound examination in your clinical practice?*

The next question addressed respondents’ opinions on whether having the ability to perform a basic ultrasound examination would improve their clinical work. Nearly all participants (N = 82; 96.5%) responded positively. A total of 1.2% selected the response “I don’t know,” and the same proportion (1.2%) gave a negative answer.

**Figure 35**

*Percentage distribution of responses to the question: Do you believe that the ability to perform a basic ultrasound examination would improve your clinical work?*

The next question asked respondents to indicate which organs they believed would benefit from ultrasound assessment in their clinical practice. The majority (77.6%; N = 66) selected vessels used for vascular access placement. More than half of the respondents indicated the lungs (N = 44; 51.8%), pleural cavities (N = 47; 55.3%), and heart (N = 45; 52.9%).
Over 40% identified the peritoneal cavity (N = 42; 49.4%) and deep veins (N = 37; 43.5%) as relevant structures. A total of 35.3% (N = 30) reported that they would use ultrasound to examine the bladder. More than 20% indicated that ultrasound would be useful for assessing the biliary ducts (N = 17; 20.0%), liver (N = 21; 24.7%), kidneys (N = 22; 25.9%), and pericardial sac (N = 23; 27.1%). Between 10% and 20% of respondents mentioned the pancreas (N = 11; 12.9%), lymph nodes and thyroid gland (each N = 15; 17.6%), and spleen (N = 16; 18.8%). The smallest proportion of respondents (N = 4; 4.7%) indicated the salivary glands.

**Figure 36**
Percentage distribution of responses to the question: *Which organs, in your opinion, would benefit from ultrasound assessment in your clinical practice?*

Respondents were then asked whether their residency program prepares or has prepared them to perform point-of-care ultrasound (POCUS) examinations. A total of 76.5% (N = 65) reported that their program did not include such preparation. An affirmative response was given by 22.4% (N = 19), while one respondent indicated “I don’t know.”

**Figure 37**
Percentage distribution of responses to the question: *Does/did your residency program prepare you to perform point-of-care ultrasound examinations?*

Respondents were then asked whether they had participated in ultrasound training courses on their own initiative. More than 80% reported having done so—35.3% (N = 30) occasionally and 45.9% (N = 39) multiple times. A total of 18.8% (N = 16) stated that they had never participated in such self-initiated training.

**Figure 38**
Percentage distribution of responses to the question: *Have you ever participated in ultrasound training on your own initiative?*

The next question concerned invasive procedures performed under ultrasound guidance. Nearly 70% of respondents (N = 58; 68.2%) reported using ultrasound during vascular access placement. A total of 16.5% (N = 14) indicated using ultrasound during thoracentesis or paracentesis, while 14.1% (N = 12) stated that they did not possess the skills required to perform such procedures under ultrasound guidance.

**Figure 39**
Percentage distribution of responses to the question: *Do you perform any invasive procedures under ultrasound guidance?*

The final question asked respondents where they had the opportunity to learn how to perform invasive procedures under ultrasound guidance. A total of 37.6% (N = 32) reported acquiring these skills through self-initiated training courses. Another 30.6% (N = 26) stated that they developed these competencies during their internship, while 28.2% (N = 24) gained such experience during residency training. Only 3.5% (N = 3) of respondents indicated that they had acquired these skills during medical school.

**Figure 40**
Percentage distribution of responses to the question: *Where did you have the opportunity to learn how to perform invasive procedures under ultrasound guidance?*
